# *Leishmania infantum* triggers lipid peroxidation and non-canonical cell death in macrophages

**DOI:** 10.3389/fimmu.2026.1891865

**Published:** 2026-10-15

**Authors:** Silke Mortelmans, Bregje Christiaenssen, Sarah Hendrickx, Geert van Loo, Tom Vanden Berghe, Guy Caljon, Andy Wullaert

**Affiliations:** 1 Cell Death Signaling Laboratory, Infla-Med Centre of Excellence, Department of Biomedical Sciences, University of Antwerp, Antwerp, Belgium; 2 Laboratory of Microbiology, Parasitology and Hygiene, Infla-Med Centre of Excellence, Department of Biomedical Sciences, University of Antwerp, Antwerp, Belgium; 3 VIB Center for Inflammation Research, VIB, Ghent, Belgium; 4 Department of Biomedical Molecular Biology, Ghent University, Ghent, Belgium; 5 Department of Internal Medicine and Pediatrics, Ghent University, Ghent, Belgium

**Keywords:** cell death, ferroptosis, gasdermin D (GSDMD), inflammasome, *Leishmania*

## Abstract

*Leishmania infantum* is a causative agent of visceral leishmaniasis and survives as an obligate intracellular parasite within macrophages by modulating host cell death pathways. While preservation of host cell viability is critical for parasite replication, infected macrophages ultimately undergo cell death, although the mechanisms remain unclear. Here, we investigated the nature of *L. infantum*-induced cell death in murine bone marrow-derived macrophages (BMDMs). Using long-term live-cell imaging, we show that *L. infantum* induces delayed cell death in lipopolysaccharide (LPS)-primed macrophages. This process occurred independently of Gasdermin D (GSDMD) and was not associated with inflammasome activation, as evidenced by the absence of caspase-1 cleavage and IL-1β secretion. Consistently, GSDMD deficiency did not affect visceral parasite burden or systemic inflammation during *L. infantum* infections in mice. Combined genetic ablation of key mediators of apoptosis (caspase-8), necroptosis (RIPK3), and inflammasome signaling (caspase-1/11) failed to protect macrophages from *L. infantum*-induced cell death, indicating that classical lytic and apoptotic pathways were dispensable. Instead, *L. infantum*-induced non-canonical macrophage death was associated with robust lipid peroxidation, a hallmark of ferroptosis. However, pharmacological inhibition of lipid peroxidation using ferrostatin-1 did not rescue cell viability, while genetic ablation of the anti-ferroptotic enzyme GPX4 enhanced lipid peroxidation without further sensitizing infected macrophages to death. These findings suggest that *L. infantum* is capable of uncoupling lipid peroxidation from cell death in macrophages. Together, our results reveal that *L. infantum* induces lipid peroxidation without triggering ferroptosis and instead drives a non-canonical form of macrophage death independent of major known pathways.

## Introduction

Leishmaniases are a group of vector-borne tropical diseases caused by protozoan parasites of the genus *Leishmania* that are transmitted when infected sand flies inoculate metacyclic promastigotes into the host skin. Depending on the infecting *Leishmania* species and the host immune response, ensuing clinical manifestations range from self-healing cutaneous leishmaniasis (CL) to a severe visceral leishmaniasis (VL) characterized by persistent fever, hepatosplenomegaly, and a high fatality rate if left untreated (1). One of the main viscerotropic parasites causing VL is *Leishmania (L.) infantum*, a zoonotic species found across the Mediterranean basin, Central Asia, and Latin America (2).

VL develops when visceralizing parasites are phagocytosed by neutrophils and monocytes, which these parasites use as Trojan horses to access macrophage phagolysosomes in which they differentiate into amastigotes and as such disseminate to the spleen, liver, and bone marrow where they persist by actively evading and suppressing host immunity (3, 4). As an obligate intracellular parasite, *Leishmania* must preserve macrophage viability to maintain its replicative niche during the persistent infection. Macrophages can undergo multiple forms of cell death in response to intracellular pathogens, including apoptosis, pyroptosis, necroptosis, and ferroptosis. While phagocytosis of apoptotic cell corpses allows immunologically silent parasite spread, lytic cell death modes eliminate the parasite’s replicative niche and attract activated immune cells to clear the pathogens. As such, the overall impact of a viscerotropic parasite such as *L. infantum* on the various signaling pathways governing these different cell death modalities will ultimately determine VL development and severity (5, 6).

Pyroptosis is a lytic cell death mode mediated by Gasdermin D (GSDMD) cleavage downstream of inflammasome activation (7) for which the role in leishmaniasis remains particularly enigmatic. Several *Leishmania* species, including *L. infantum*, were shown to activate the Nlrp3 inflammasome in macrophages (8–16). However, depending on the infecting species, Nlrp3 activation had detrimental or protective impacts on leishmaniasis pathology. While the inflammasome was important for restricting cutaneous *L. braziliensis* or *L. amazonensis* infections (9, 13–15), it did not influence or even aggravated pathology upon cutaneous *L. mexicana* or *L. major* infections (10–12, 16). Despite these *Leishmania* species-specific effects of Nlrp3 inflammasome activation, studies demonstrated increased susceptibility of GSDMD-deficient mice to cutaneous *L. amazonensis*, *L. braziliensis*, *L. major* and *L. mexicana* infections, supporting a more broadly acting protective effect of GSDMD (15, 16). However, while inflammasome signaling was shown to protect mice from an intraperitoneal infection with *L. infantum chagasi* (9), the role of downstream GSDMD-mediated pyroptosis during an *L. infantum* infection either in mice or in macrophages has not been explored.

Here, we show that GSDMD deficiency did not impact on *L. infantum* infection kinetics or systemic inflammation in mice. In addition, GSDMD-deficient macrophages still exhibited cell death upon *L. infantum* infection. Infections in macrophages lacking several cell death signaling mediators ruled out caspase-8-dependent apoptosis, RIPK3-dependent necroptosis as well as all inflammasome-dependent pathways as culprits for this *L. infantum*-induced GSDMD-independent cell death. Moreover, although *L. infantum* induced lipid peroxidation in macrophages, pharmacological inhibitory and genetic boosting approaches could not causally link this response to ferroptotic cell death. In conclusion, our analyses show that *L. infantum*-infected macrophages remain capable of executing cell death even in the absence of several key signaling intermediates, although the exact nature of this cell death mode remains unclear.

## Materials and methods

### Ethics statement

All animal experiments were performed according to institutionally approved protocols according to national (Belgian Laws 14/08/1986 and 22/12/2003, Belgian Royal Decree 06/04/2010) and European (EU Directives 2010/63/EU, 86/609/EEG) animal regulations. Animal protocols were reviewed and approved by the Ethical Committee Animal Experimentation – University of Antwerpen - Faculty of Pharmaceutical, Biomedical and Veterinary Sciences (permit number LA1100465) with approval ID 2022-61. All necessary efforts were made to minimize suffering of the animals.

### Mice

The Gsdmd^-/-^ (17), Casp1/11^−/−^ (18), Casp8^−/−^Ripk3^−/−^ (19–22), and Gpx4^FL^ (23) genetic mouse models used in this study were described previously. Fcgr1Cre transgenic mice (B6-Fcgr1tm3Ciphe) (24) were provided by Dr. Bernard Malissen (Centre d’Immunologie de Marseille-Luminy, France). All mice used in this study were either generated on C57BL/6 background or backcrossed at least ten generations to C57BL/6J background. More complex genetic mouse models were generated by intercrossing the above lines. All mice were bred in-house in the Specific Pathogen Free (SPF) facility at the University of Antwerpen. In all experiments, mice were housed in a 12h light - 12h dark cycle and were fed autoclaved standard rodent feed (Ssniff, Soest, Germany) *ad libitum* with free access to drinking water. All mice used in *in vivo* infection experiments were age-matched littermate males that were assigned to experimental groups according to genotype.

### Parasite culture

The *L. infantum* strain MHOM/FR/96/LEM3323 used in all experiments was kindly provided by CNRL (Montpellier, France). The strain was transfected to express PpyRE9 and DsRed reporter genes integrated into the 18S rDNA locus for bioluminescent and fluorescent detection (25, 26). Promastigotes were cultured at 25 °C in hemoflagellate-modified minimal essential medium (HOMEM) supplemented with 10% heat-inactivated fetal bovine serum (FBS, Gibco), 2.5% Pen/Strep (100 U/mL, Gibco), 4 mM L-glutamine (Gibco), 16 mM NaHCO_3_ (Sigma-Aldrich), 40 mg/L adenine (Sigma-Aldrich), 3 mg/L folic acid (Sigma-Aldrich), 2 mg/L D-biotin (Sigma-Aldrich) and 5 mg/L hemin (Sigma-Aldrich). 150 μg/mL hygromycin (Jena Bioscience) and 100 μg/mL nourseothricin (Jena Bioscience) were added to select for parasites expressing PpyRE9 and DsRed, respectively. Passage number was kept below 20 to ensure parasite virulence. All experiments were performed using stationary-phase promastigotes.

### Bone marrow derived macrophages (BMDM) differentiation and infection

BMDMs were generated by culturing bone marrow cells in Iscove’s modified Dulbecco’s medium (IMDM) containing 10% heat-inactivated FBS (Gibco), 30% L929 cell-conditioned medium, 1% nonessential amino acids (Gibco), 1% penicillin/streptomycin (100 U/mL, Gibco) and 2 mM L-glutamine (Gibco) at 37 °C in a humidified atmosphere containing 5% CO_2_. Six days after culturing, cells were seeded in 12-well plates (800,000 cells per well) or 96-well plates (40,000 cells per well) in RPMI 1640 medium containing 100 ng/mL murine M-CSF (VIB Protein Core), 10% FBS and 1% nonessential amino acids. The next day, selected wells were stimulated with 100 ng/mL lipopolysaccharide (LPS) from *Salmonella Minnesota* (Invivogen) for 4 hours at 37 °C. Afterwards, cells were infected with *L. infantum* using a multiplicity of infection (MOI) of 10. Untreated BMDMs served as the baseline control. Nigericin (6.7 µM) and ATP (5 mM) were used as positive controls for pyroptosis, while cumene hydroperoxide (CuOOH, 1 mM) was used as a positive control for ferroptosis. CuOOH co-treated with ferrostatin-1 (Fer-1, 25 µM) served as a negative control for ferroptosis. For infection, rosettes were disrupted by passing the parasites through a 25G needle. Parasite density was determined by counting using a KOVA counting chamber.

### Mouse *Leishmania infantum* infections

Mice were divided in experimental groups based on genotype and kept in individually ventilated cages in SPF conditions with food for laboratory rodents (Carfil) and water *ad libitum* for at least 5 days before the start of the experiment. Stationary-phase promastigotes were centrifuged at 1500 g for 10 min and resuspended in sterile RPMI medium to a density of 1×10^9^ promastigotes/mL. Mice were intravenously injected with 10^8^ parasites in 100 μL in the lateral tail vein. Parasite loads were monitored weekly using the IVIS Spectrum imaging system (Perkin Elmer). Mice were injected intraperitoneally with 150 mg/kg D-luciferin (Beetle Luciferin Potassium Salt, Promega) and imaged for 15 minutes under 2.5% isoflurane anesthesia. Luminescent signals were quantified as relative luminescence units (RLU) using the LivingImage v4.3.1 software.

### Cytokine analyses

Cell-free supernatant from BMDMs were collected and diluted 1:3. Serum samples were prepared from fresh blood, which was kept at room temperature for 30 minutes to clot followed by cold centrifugation for 10 minutes at 1400 g. IL-1β cell supernatant levels as well as IFN-γ serum levels were determined by magnetic bead-based multiplex assay using Luminex technology (Bio-Rad) according to the manufacturer’s instructions. IL-1β serum levels were determined by the Mouse IL-1 beta/IL-1F2 Quantikine HS ELISA Kit (R&D systems) according to the manufacturer’s protocol. Cytokines from cell supernatant were expressed as concentration per mL of serum. Cytokines from serum were normalized to the uninfected controls as fold change.

### Olink analysis

Serum inflammatory protein profiling was performed using a proximity extension assay with the Target 48 Mouse Cytokine panel (Olink #93400) according to the manufacturer’s instructions. Briefly, 1 µL of serum from fresh aliquots stored at −80 °C was thawed and incubated for 16h at 4 °C in an incubation mix containing cytokine-specific antibody pairs, each coupled to forward and reverse probes. Extension of the complementary probes occurred on a C1000 Touch Thermal Cycler (Bio-Rad Laboratories #CT039644) and was possible only when both antibodies corresponding to a single cytokine were in close proximity, binding to neighboring epitopes on the target cytokine. For detection, an IFC 48.48 microfluidic chip (Olink #93007) was primed and loaded with samples and probes, and real-time PCR was performed on a Signature Q100 instrument (Olink^®^). PCR data analysis was conducted using the Signature Q100 PCR Analysis software (Olink^®^), with automatic (global) Ct threshold determination set. Data processing, quality control, and determination of absolute concentrations were performed using the Olink^®^ NPX Signature software (v1.16.0).

### Western blotting

BMDMs were incubated with cell lysis buffer (2 mM Tris-HCl (pH 7.4), 20 mM NaCl, 0.1% NP-40) and were denatured in Laemmli buffer by boiling for 10 min. Proteins were separated by SDS-PAGE and transferred to nitrocellulose membranes. Blocking, antibody incubations, and washing of the membrane were done in TBS supplemented with 0.1% Tween 20 (v/v) and 5% (w/v) non-fat dry milk. Immunoblots were incubated overnight with primary antibody against Caspase-1 (1:1000; AdipoGen Life Sciences; catalog number AG20B-0042-C100), GSDMD (1:1000; Abcam; catalog number ab209845), Caspase-3 (1:1000; Cell Signaling Technology; catalog number 9662S), cleaved Caspase-3 (1:1000; Cell Signaling Technology; catalog number 9661), Caspase-8 (1:1000; Enzo life sciences, catalog number ALX-804-447-C100), cleaved Caspase-8 (1:1000; Cell Signaling Technology; catalog number 8592), GPX4 (1:1000; Abcam; catalog number ab125066) or β-actin (1:5000; Santa Cruz; catalog number sc-47778). Appropriate horseradish peroxidase-conjugated secondary antibodies (Jackson ImmunoResearch Laboratories; catalog numbers 111-035-144, 115-035-146) were used to detect primary antibodies. Proteins of interest were visualized by the enhanced SuperSignal West Pico Chemiluminescent Substrate (Thermo Scientific) or SuperSignal West Femto Maximum Sensitivity Substrate (Thermo Scientific).

### Cell death assay

Monitoring real-time cellular membrane integrity was performed with a Spark Cyto 600 plate reader (TECAN) using the non-cell permeable SYTOX Green Nucleic Acid Stain (250nM, Invitrogen). The number of positive cells was calculated with the ImageJ Fiji software. These numbers were normalized to a 100% cell count control achieved by treating cells with 1% Triton-X-100. LDH release was determined using the Cytotox 96 Non-Radioactive Cytotoxicity Assay (Promega), according to the manufacturer’s instructions. After 96 hours of infection, culture supernatants from BMDMs were collected and incubated with the substrate for 30 min in the dark. After addition of stop solution, absorbance was measured at 490 nm using the PerkinElmer EnVision 2103 Multilabel Reader and normalized to a 100% cell death control achieved by treating with lysis buffer for 30 min.

### Lipid peroxidation assay

Lipid peroxidation was evaluated using the Image-iT Lipid Peroxidation Kit, (Invitrogen) according to the manufacturer’s instructions. Infected BMDMs were stained with 10 µM C11-BODIPY™ 581/591 at 37 °C for 30 min and the cells were washed with 1× PBS. Live/Dead staining was performed with Fixable viability dye eFluor 450 (1:1000; Invitrogen) at 4 °C for 20 min. After washing, samples were acquired on a NovoCyte Quanteon Flow Cytometer (Agilent) or a MACSQuant Analyzer 10 Flow Cytometer (Miltenyi Biotec). Data were analyzed using FlowJo v.10 (BD Bioscience).

### ROS detection

Intracellular ROS was detected using the CellROX Deep Red Reagent (Invitrogen). Infected BMDMs were stained with 200 nM CellROX Deep Red for 30 min at 37 °C and washed with 1× cold PBS. Live/Dead staining was performed with Fixable viability dye eFluor 450 (1:1000; Invitrogen) at 4 °C for 20 min. After washing, samples were acquired on a NovoCyte Quanteon Flow Cytometer (Agilent). MFIs were extracted after gating on live cells using FlowJo v.10 (BD Bioscience), and were expressed as fold induction relative to untreated BMDMs.

### Statistical analyses

GraphPad Prism10.1 software was used for data analysis. All data are presented as means with SD. Data were analyzed by applying either unpaired student *t*-tests or two-way ANOVA with Sidak’s multiple comparisons in case of interaction with a time variable. For the *in vivo* study, mixed effects models were used to analyze repeated measures data, and additional student *t*-tests were performed to analyze differences between genotypes at individual time points. *p < 0.05; **p < 0.01; ***p < 0.001.

## Results

### *Leishmania infantum* induces cell death in LPS-primed murine macrophages

To understand how cellular macrophage responses could potentially contribute to the parasitic life cycle during VL, we infected primary murine bone marrow derived macrophages (BMDMs) with the VL-causative parasite *L. infantum* after which we monitored the cells by long-term real-time imaging. Parasitic expression of a dsRed fluorescent marker revealed an efficient infection of nearly all BMDMs starting from 24h post-infection and retaining this infection rate throughout the observation period of 96h (**Figures 1A, B**). Besides infection efficiency, addition of the cell impermeable fluorescent dye Sytox Green enabled to simultaneously detect loss of plasma membrane integrity, with Sytox Green positivity suggesting ongoing cell death. Sytox Green positive *L. infantum*-infected BMDMs could be detected from 48h post-infection onward and were omnipresent by 72h post-infection (**Figures 1A, C**). However, uninfected BMDMs displayed a very similar Sytox Green staining kinetic (**Figures 1A, C**), indicating that these unchallenged primary macrophages did not survive long-term culturing beyond 48h. This observation precluded us from drawing sound conclusions about the specific cytotoxic effects of *L. infantum* infection in otherwise naïve macrophages.

**Figure 1:**
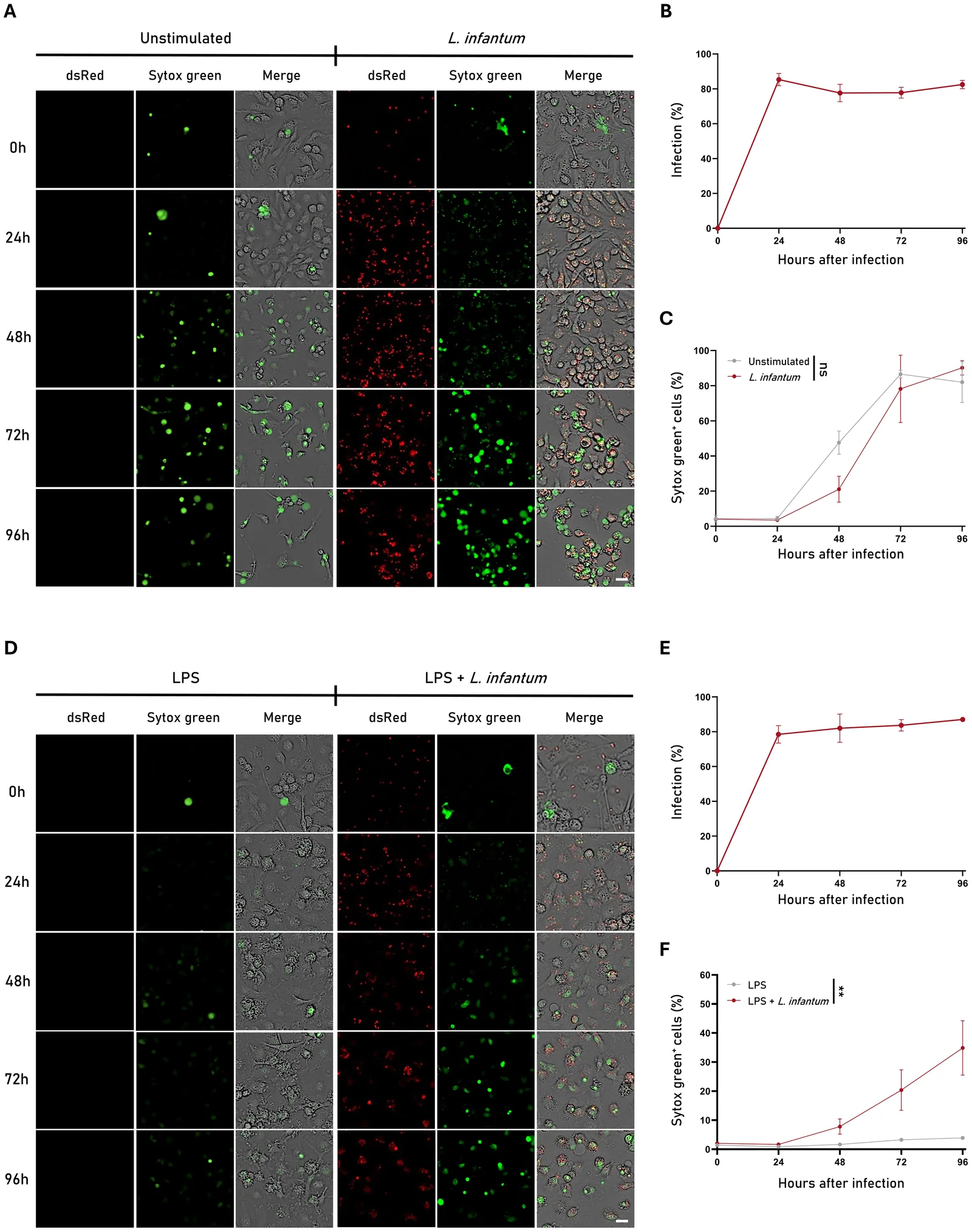
*Leishmania infantum* induces cell death in LPS-primed macrophages. **(A–C)** Naïve BMDMs (n=3) were either non-infected or infected with MOI 10 of the LEM3323 WT^PpyRE9/DsRed^ *L. infantum* strain expressing DsRed in the presence of SYTOX Green. **(A)** Representative pictures of real-time monitoring of DsRed and SYTOX Green fluorescence at indicated time periods, showing infection efficiency and plasma membrane permeabilization, respectively, **(B)** quantification of infection kinetics based on DsRed positivity, **(C)** quantification of cell permeability based on SYTOX Green positivity. **(D–F)** BMDMs (n=3) were treated with 100 ng/mL LPS for 4h and were then left non-infected or infected with MOI 10 of the LEM3323 WT^PpyRE9/DsRed^ *L. infantum* strain expressing DsRed in the presence of SYTOX Green. **(D)** Representative pictures of real-time monitoring of DsRed and SYTOX Green fluorescence at indicated time periods, showing infection efficiency and plasma membrane permeabilization, respectively, **(E)** quantification of infection kinetics based on DsRed positivity, **(F)** quantification of cell permeability based on SYTOX Green positivity. Data in **(B, C)** and **(E, F)** represent means ± SD of BMDMs taken from three individual mice. Statistics were analyzed by unpaired student t-test at 96 hours post infection. ns, not significant; ** p<0.01.

Therefore, to prevent spontaneous death of non-infected control macrophages, we next assessed the effects of *L. infantum* infection in BMDMs that were primed beforehand with lipopolysaccharide (LPS). As LPS-induced TLR4 signaling is known to prolong primary macrophage survival (27), we reasoned that an LPS challenge would render BMDMs more resilient during long-term culturing. Indeed, we observed unchanged cellular morphology and hardly any Sytox Green positive LPS-treated BMDMs throughout a period of 96h real-time imaging (**Figure 1D**), indicating that LPS effectively preserved long-term macrophage viability. Importantly, the pro-inflammatory environment created by the prior LPS challenge did not affect the ability of *L. infantum* to infect or persist in BMDMs, as about 80% of the LPS-treated BMDMs were infected by *L. infantum* from 24h throughout 96h post-infection (**Figure 1E**). Interestingly, compared to uninfected LPS-treated cells, we observed a statistically significant cytotoxic effect of *L. infantum* in LPS-primed BMDMs as indicated by the loss of plasma membrane integrity in about 35% of macrophages by 96h post-infection (**Figures 1E, F**). Thus, while drawing conclusions in naïve conditions was not possible, our observations showed that the VL-causative *L. infantum* could induce cell death in primary macrophages in LPS pre-treated conditions.

### *Leishmania infantum*-induced cell death in LPS-primed macrophages is independent of Gasdermin D and is not associated with inflammasome activity

Besides inducing a transcriptional response aimed at preventing apoptotic cell death, treatment of macrophages with LPS can conversely sensitize to pyroptotic cell death when confronted with an appropriate stimulus triggering the Nlrp3 inflammasome. Pyroptosis is a form of cell death resulting from a priming signal such as LPS that upregulates Nlrp3 expression and a second activation signal that subsequently activates the Nlrp3 inflammasome (7). We thus hypothesized that the above observed death of LPS-primed *L. infantum*-infected macrophages constituted pyroptosis, with *L. infantum* providing the secondary Nlrp3 activation trigger after LPS priming. To test this hypothesis, because plasma membrane permeabilization during Nlrp3-mediated pyroptosis is caused by the pore-forming protein Gasdermin D (GSDMD), we monitored Sytox Green permeability kinetics in LPS-primed *L. infantum*-infected Gsdmd^-/-^ BMDMs. However, LPS-treated Gsdmd^-/-^ BMDMs responded similarly to an *L. infantum* infection as compared to LPS-treated BMDMs from Gsdmd^+/+^ littermates. Both genotypes were infected with *L. infantum* with similar efficiencies (**Figures 2A, B**), and the parasite caused comparable rates of plasma membrane permeabilization in LPS-primed Gsdmd^-/-^ and Gsdmd^+/+^ macrophages (**Figures 2C, D**). The latter analyses confirmed a statistically significant induction of cell death by *L. infantum* in LPS-treated BMDMs of both genotypes, but Gsdmd^-/-^ macrophages showed parallel cell death kinetics as compared to Gsdmd^+/+^ macrophages (**Figures 2C, D**).

**Figure 2:**
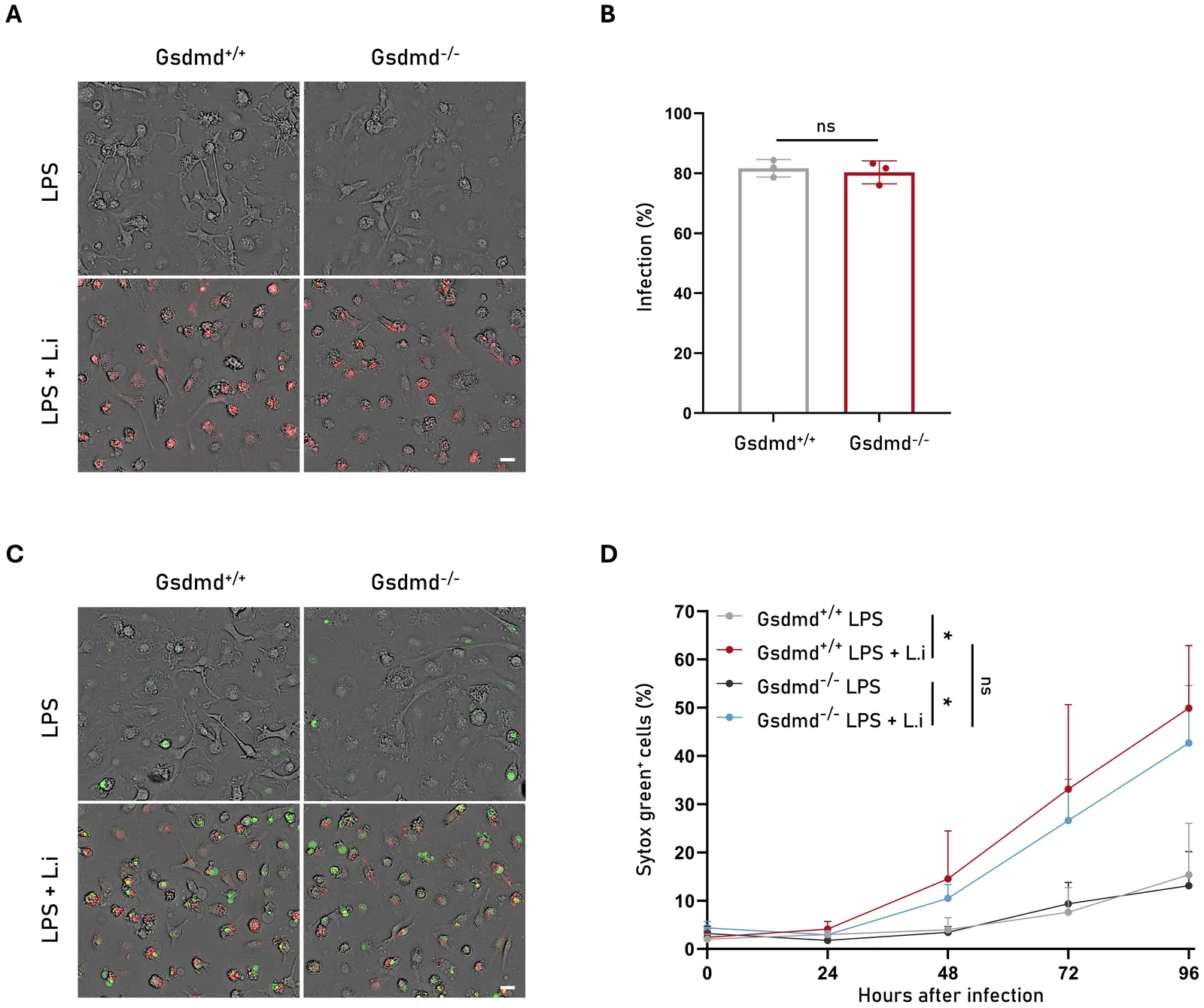
*Leishmania infantum*-induced cell death in LPS-primed macrophages is independent of GSDMD. **(A–D)** Gsdmd^+/+^ and Gsdmd^-/-^ BMDMs (n=3) were treated with 100 ng/mL LPS for 4h and were then left non-infected or infected with MOI 10 of the LEM3323 WT^PpyRE9/DsRed^ *L. infantum* strain expressing DsRed for indicated time periods in the presence of SYTOX Green. **(A)** Representative pictures and **(B)** quantification of DsRed fluorescence showing infection efficiency. **(C)** Representative pictures and **(D)** quantification of SYTOX Green fluorescence showing cell permeability. Data in **(B, D)** represent means ± SD of BMDMs taken from three individual mice per genotype. Statistics were analyzed by unpaired student t-test at 96 hours post infection. ns, not significant; ** p<0.01.

The above observation that *L. infantum* provoked cell death independently of GSDMD suggested that GSDMD was not activated. To evaluate GSDMD activation we performed immunoblotting analyses assessing its proteolytic cleavage into the N-terminal p30 fragment that holds the cytotoxic pore-forming activity. Neither at 72h nor at 96h post-infection we could detect this p30 fragment in LPS-primed *L. infantum*-infected BMDMs (**Figure 3A**). Although we observed smaller molecular weight fragments in Gsdmd^+/+^ macrophages that were not present in Gsdmd^-/-^ cells, these smaller GSDMD products did not correspond to the p30 fragment of the positive control and they were also present in samples from non-infected LPS-treated Gsdmd^+/+^ macrophages (**Figure 3A**). Thus, these immunoblotting analyses did not reveal GSDMD cleavage specifically induced by *L. infantum* infection. To further evaluate whether an inflammasome pathway leading to GSDMD activation was present upon *L. infantum* infection, we assessed caspase-1 cleavage as a marker for inflammasome activity. However, immunoblotting did not reveal *L. infantum*-induced generation of the active p20 subunit of caspase-1 in either of the GSDMD genotypes (**Figure 3B**), suggesting a lack of inflammasome activation. Nevertheless, as a more quantitative parameter of inflammasome activity, we measured the presence of IL-1β in the cell culture supernatants by means of a sensitive ELISA. These analyses confirmed that *L. infantum* did not induce inflammasome activation, as infecting LPS-primed macrophages with the parasite for either 72h or 96h did not increase IL-1β release as compared to LPS treatment only (**Figures 3C, D**). We also did not detect either GSDMD cleavage, caspase-1 activation or IL-1βsecretion at 24h or 48h post-infection (**Supplementary Figure S1**), excluding transient inflammasome signaling in *L. infantum*-infected BMDMs. Taken together, these caspase-1 cleavage and IL-1β secretion analyses indicated that *L. infantum* did not elicit detectable inflammasome activity in LPS-primed BMDMs, consistent with our observation that the downstream pyroptosis effector GSDMD was neither activated nor involved in *L. infantum*-induced cell death in these LPS-treated macrophages. Notably, in contrast to IL-1β, *L. infantum*-infected BMDMs released LDH in the cell culture medium irrespective of their GSDMD genotype (**Figure 3E**). This indicated that *L. infantum*-infected BMDMs underwent a lytic cell death mode independent from inflammasome signaling or GSDMD.

**Figure 3:**
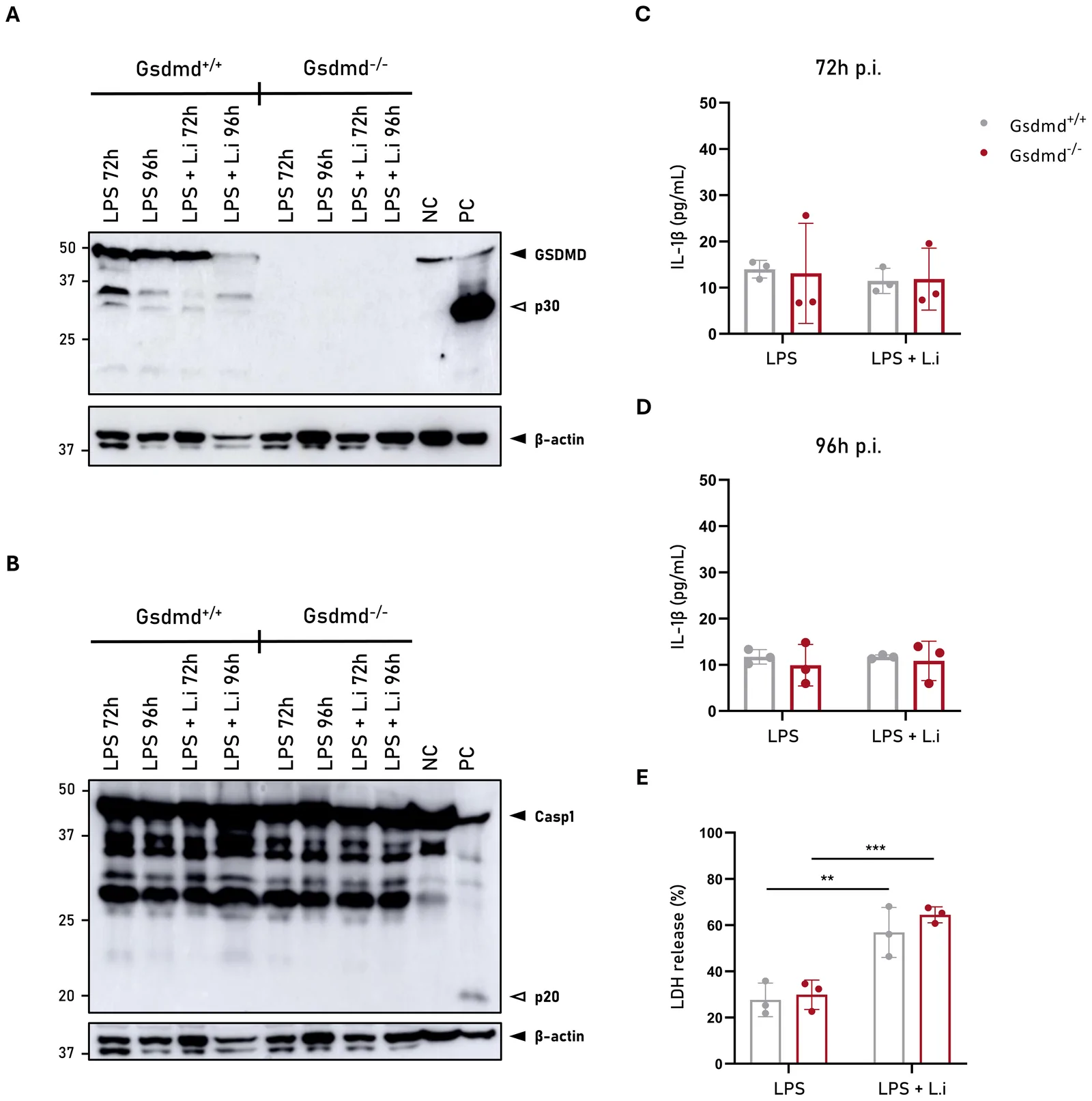
LPS-primed macrophages do not show inflammasome activity upon infection with *Leishmania infantum*. **(A–E)** Gsdmd^+/+^ and Gsdmd^-/-^ BMDMs were treated with 100 ng/mL LPS for 4h and were then left non-infected or infected with MOI 10 of the LEM3323 WT^PpyRE9/DsRed^ *L. infantum* strain expressing DsRed for indicated time periods. **(A, B)** Cleavage of **(A)** GSDMD or **(B)** caspase-1 were assessed by immunoblotting. BMDMs treated with only LPS or with LPS/ATP served as negative control (NC) and positive control (PC), respectively. **(C, D)** IL-1β secretion was measured in cell supernatants at **(C)** 72h or **(D)** 96h post-infection. **(E)** LDH release was measured in cell supernatants at 96h post-infection. Data in **(C–E)** represent means ± SD of BMDMs taken from three individual mice per genotype. Statistics were analyzed by unpaired student t-test. ns, not significant; *p<0.05; **p<0.01; ***p<0.001.

### Parasite infection and clearance kinetics as well as cytokine responses during *Leishmania infantum*-induced visceral leishmaniasis in mice are not affected by Gasdermin D

Although the above BMDM experiments did not reveal a role for GSDMD in regulating *L. infantum*-induced cell death, these *ex vivo* differentiated macrophages may not be representative for tissue-resident macrophages such as Kupffer cells that are the initial *in vivo* host cells for *L. infantum* (28). Moreover, a CL study using *L. mexicana* showed that also neutrophils utilize GSDMD responses to modulate *in vivo* leishmaniasis pathology (16). Therefore, to investigate the involvement of GSDMD-mediated cell death in the *L. infantum* life cycle in a physiological context, we monitored *in vivo L. infantum* loads in Gsdmd^-/-^ and Gsdmd^+/-^ littermates during a 12-week systemic VL infection. For this purpose, we took advantage of a bioluminescent *L. infantum* strain enabling the weekly quantification and localization of parasites by bioluminescent imaging (BLI). In both Gsdmd^-/-^ and Gsdmd^+/-^ mice, we observed comparable hepatic *L. infantum* infection profiles reaching their peaks at three weeks post-infection (wpi) and showing subsequent declines of the parasite loads leading to clearance by 8 wpi (**Figures 4A, B**), although it should be noted that livers of Gsdmd^-/-^ mice displayed a temporarily increased parasite burden at 4 wpi (**Figure 4B**). Possibly owing to the *Leishmania*-resistant nature of their C57Bl/6 genetic backgrounds, neither Gsdmd^-/-^ nor Gsdmd^+/-^ cohorts showed consistent parasitic invasion of the bone marrow, and no significant differences could be observed between bone marrow infection rates between both genotypes (**Figures 4A, C**). Thus, tracking *L. infantum* over time showed similar kinetics of parasitic colonization, spreading and elimination in Gsdmd^-/-^ and Gsdmd^+/-^ mice. Despite these equal parasite loads throughout the infection, we next evaluated whether full body GSDMD deficiency affected systemic inflammatory responses provoked by the parasites. For this purpose, we first performed an Olink analysis to quantify a broad panel of cytokines, chemokines and other inflammatory mediators in serum of Gsdmd^+/-^ mice before infection as well as at various time points during the *L. infantum* infection. These analyses identified multiple inflammatory factors that were significantly upregulated in serum of *L. infantum*-infected mice relative to their serum levels before the infection (**Figure 4D**). In addition, these analyses showed that systemic inflammatory responses in these *L. infantum*-infected mice were most prominent at 6 wpi (**Figure 4D**). Based on these broad serum kinetic analyses in the Gsdmd^+/-^ cohort, we then selected individual cytokines for ELISA quantification in serum of *L. infantum*-infected Gsdmd^-/-^ and Gsdmd^+/-^ mice at 6 wpi. However, most cytokines showing significant fold inductions by Olink analyses circulated at absolute serum concentrations that were undetectable by conventional ELISA (data not shown). In contrast, both IL-1β and IFNγ showed detectable increased serum levels at 6 wpi *L. infantum*-infected Gsdmd^-/-^ and Gsdmd^+/-^ mice, yet these cohorts showed no statistically significant differences in circulating IL-1β or IFNγ levels at this timepoint (**Figures 4E, F**). Taken together, while our Olink serum analyses demonstrated a wide-ranging systemic inflammatory response throughout the course of an *L. infantum* infection in mice, we could not detect influences of GSDMD on selected serum cytokines present in this response. Overall, our data showing a lack of GSDMD effects on an *in vivo L. infantum* infection are in line with our *ex vivo* macrophages observations showing that GSDMD did not play a role in *L. infantum*-induced cell death.

**Figure 4:**
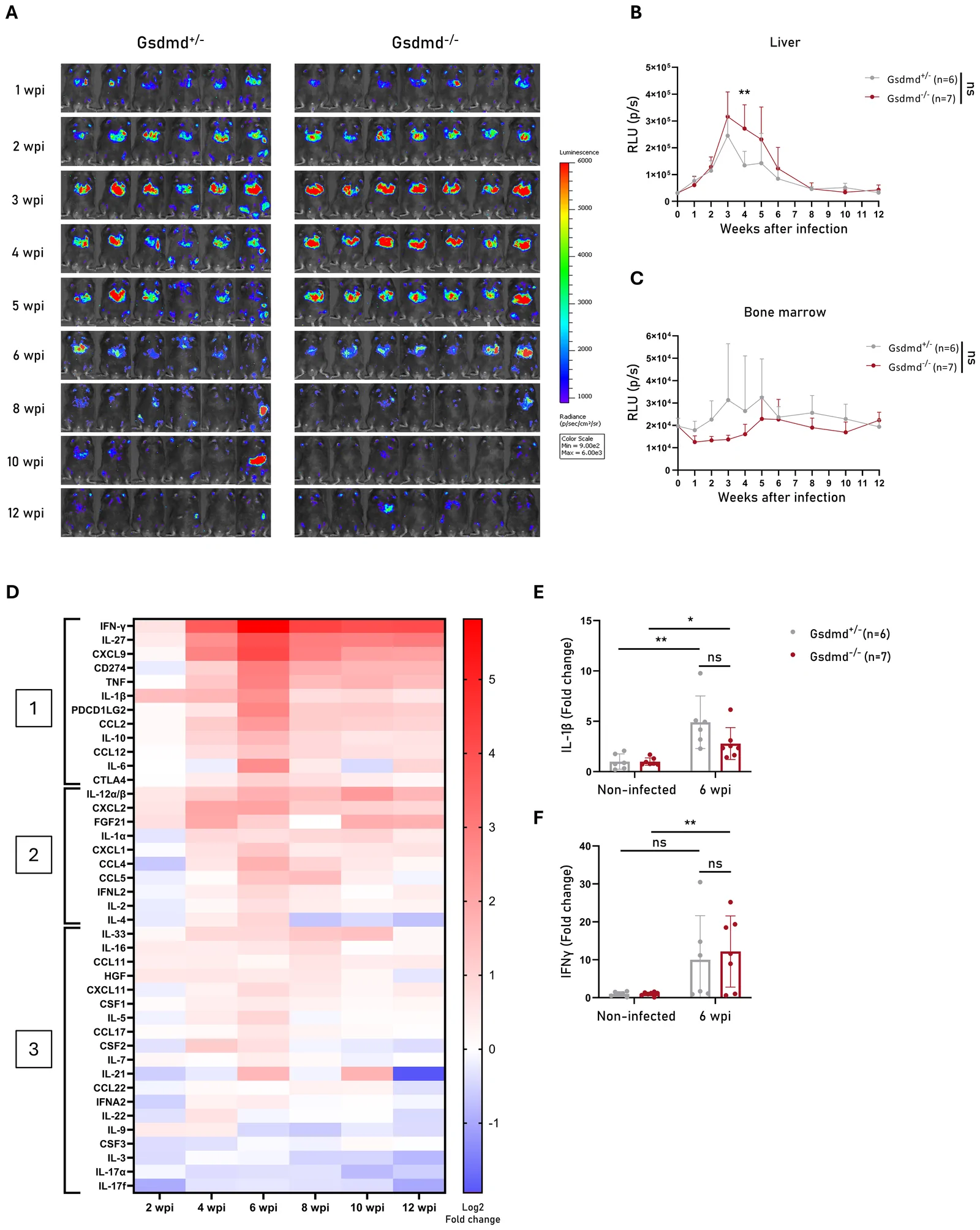
GSDMD does not impact on Leishmania infantum-induced visceral leishmaniasis in mice. **(A–F)** Gsdmd^+/-^ (n=6) and Gsdmd^-/-^ (n=7) littermates were infected intravenously with 10^8^ *L. infantum* LEM3323 WT^PpyRE9^ promastigotes and were monitored for 12 weeks. **(A)** *In vivo* bioluminescent imaging (BLI) pictures of individual mice at indicated weeks post-infection; with **(B, C)** BLI quantification in **(B)** the liver and **(C)** the bone marrow at indicated weeks post-infection. **(D)** Heatmap showing Olink analyses of inflammatory factors in serum of *L. infantum* infected Gsdmd^+/-^ mice (n=6) at indicated weeks post-infection. Log fold inductions are shown as compared with respective serum levels before infection. Statistical analyses categorized inflammatory factors in the following three clusters (1): statistically significant upregulation across the time course of infection, (2) statistically significant upregulation only at 6 weeks post-infection, or (3) no statistically significant changes. **(E, F)** Concentrations of **(E)** IL-1β and **(F)** IFNγ in serum of *L. infantum* infected Gsdmd^+/-^ (n=6) and Gsdmd^-/-^ (n=7) littermates before as well as 6 weeks post-infection measured by ELISA. Data in **(B, C, E, F)** represent means ± SD of individual mice per genotype. Whole time course statistics for **(B, C)** indicated next to the genotype legends were analyzed by mixed effects models for repeated measures, while individual timepoints in **(B, C)** indicated within the graphs were compared using unpaired student t-test. Statistics in **(E, F)** were analyzed by unpaired student t-test. ns, not significant; * p<0.05; ** p<0.01.

### *Leishmania infantum*-induced cell death in LPS-primed macrophages is independent of caspase-8, RIPK3, and canonical as well as non-canonical inflammasome signaling

Given the GSDMD-independent cell death of LPS-primed BMDMs upon *L. infantum* infection, we sought to identify the alternative cell death mode induced by *L. infantum* in these cells. Several studies showed that GSDMD deficiency allows the initiation of backup cytotoxic pathways, including caspase-8 and caspase-3 mediated apoptotic signaling that ultimately provokes a pyroptotic lytic cell death through Gasdermin E (29, 30). Therefore, we assessed caspase-8 and caspase-3 cleavage to evaluate potential activation of this pathway in LPS-primed *L. infantum*-infected Gsdmd^-/-^ BMDMs. *L. infantum* did not cause increased caspase-8 or caspase-3 cleavage in LPS-primed Gsdmd^-/-^ BMDMs as compared to LPS alone (**Figure 5A**), suggesting that GSDMD deficient conditions did not potentiate this compensatory pathway. However, we did observe a moderately increased activation of both caspase-8 and caspase-3 in LPS-primed *L. infantum*-infected Gsdmd^+/+^ BMDMs versus LPS control cells (**Figure 5A**), and analyses at earlier timepoints detected caspase-8 and caspase-3 activation at 24h and 48h post-infection, respectively (**Figure 5B**). We therefore examined the possibility that *L. infantum* induced caspase-8 dependent apoptotic cell death in LPS-primed macrophages. Given the embryonic lethality of caspase-8-deficient mice (21, 22), we utilized BMDMs from mice additionally deficient in RIPK3, thereby also precluding RIPK3-dependent necroptotic cell death. Moreover, to avoid relaying of cell death signaling to inflammasome-mediated pyroptosis, we used BMDMs from mice additionally deficient in both caspase-1 and caspase-11. LPS-primed Casp8^-/-^Ripk3^-/-^Casp1/11^-/-^ BMDMs were equally well infected by *L. infantum* as Casp8^+/+^Ripk3^+/+^Casp1/11^+/+^ BMDMs (**Figures 5C, D**). Nevertheless, LPS-primed macrophages of both genotypes showed identical kinetics of plasma membrane permeabilisation upon *L. infantum* infection (**Figures 5E, F**), indicating that the susceptibility of macrophages to *L. infantum*-induced death was not altered by the combined absence of caspases-8, -1, -11 as well as RIPK3. Together, this suggests that *L. infantum* does not kill LPS-primed macrophages through either caspase-8-dependent apoptosis, RIPK3-mediated necroptosis, or pyroptosis induced by caspase-1-dependent canonical or caspase-11-dependent non-canonical inflammasome activation.

**Figure 5:**
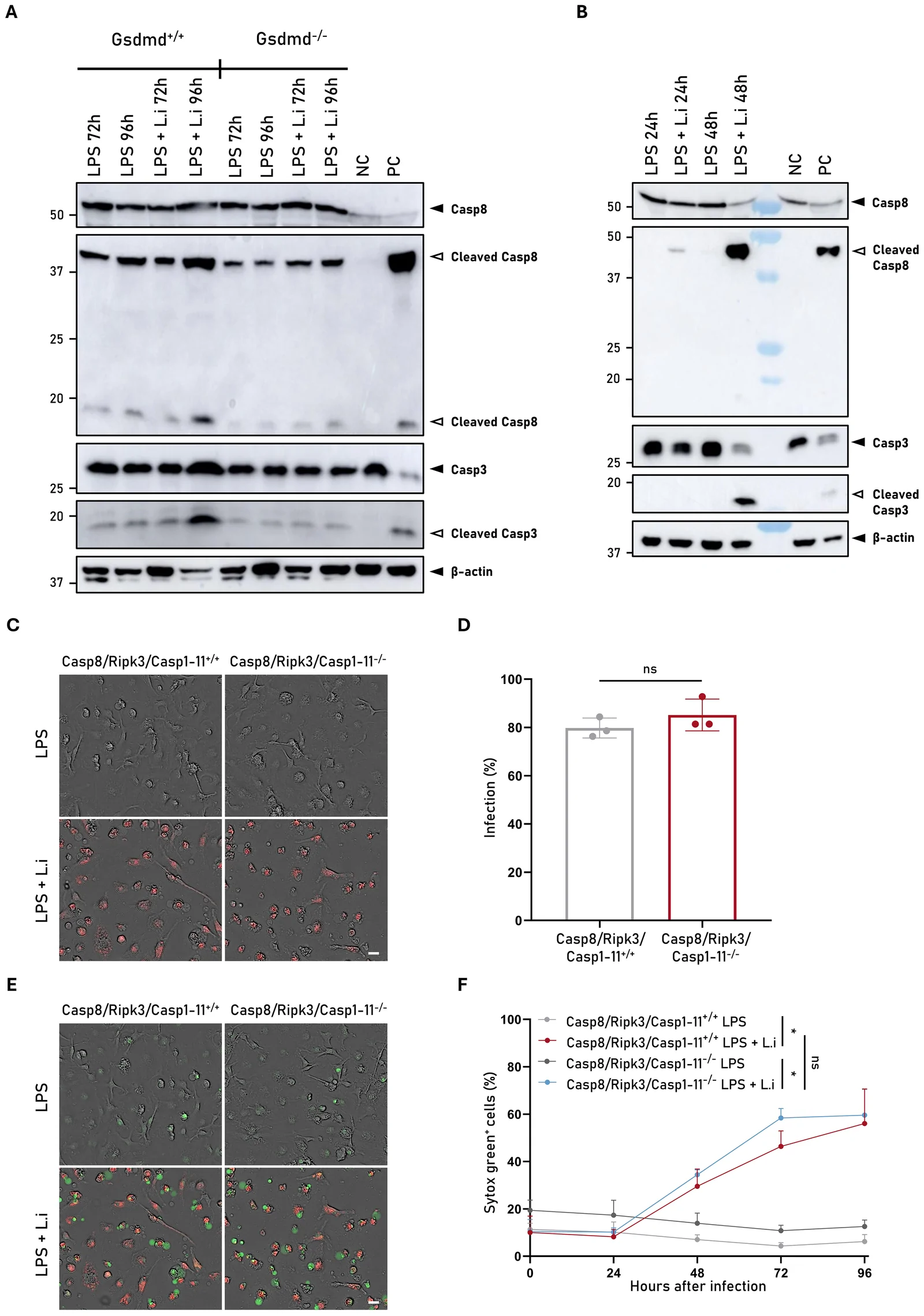
*Leishmania infantum*-induced cell death in LPS-primed macrophages is independent of caspase-8, RIPK3, as well as caspase-1 and -11. **(A, B)** Gsdmd^+/+^ and Gsdmd^-/-^ BMDMs were treated with 100 ng/mL LPS for 4h and were then left non-infected or infected with MOI 10 of the LEM3323 WT^PpyRE9/DsRed^ *L. infantum* strain expressing DsRed for indicated time periods, after which cleavage of caspase-8 and caspase-3 were assessed by immunoblotting. **(C–F)** Casp1/11^+/+^Casp8^+/+^Ripk3^+/+^ and Casp1/11^-/-^Casp8^-/-^Ripk3^-/-^ BMDMs (n=3) were treated with 100 ng/mL LPS for 4h and were then left non-infected or infected with MOI 10 of the LEM3323 WT^PpyRE9/DsRed^ *L. infantum* strain expressing DsRed for indicated time periods in the presence of SYTOX Green. **(C)** Representative pictures and **(D)** quantification of DsRed fluorescence showing infection efficiency. **(E)** Representative pictures and **(F)** quantification of SYTOX Green fluorescence showing cell permeability. Data in **(D, F)** represent means ± SD of BMDMs taken from three individual mice per genotype. Statistics in **(F)** were analyzed by unpaired student t-test at 96 hours post infection. ns, not significant; * p<0.05.

### *Leishmania infantum* infection of LPS-primed macrophages causes lipid peroxidation that does not contribute to cell death

After excluding involvement of the main apoptotic, necroptotic and pyroptotic cell death pathways, we investigated whether *L. infantum* killed LPS-primed macrophages through ferroptotic cell death. Ferroptosis is a mode of cell death specifically characterized by the accumulation of reactive lipid peroxides (31). We therefore assessed lipid peroxidation levels in LPS-primed *L. infantum*-infected BMDMs by making use of the BODIPY-C11^OX^ fluorescent probe (32). Interestingly, *L. infantum* infection in LPS-primed BMDMs resulted in the appearance of a BODIPY-C11^OX^-positive macrophage population that was absent in uninfected BMDMs treated solely with LPS (**Figure 6A**). At 96h post-infection, LPS-primed *L. infantum*-infected BMDMs comprised a significantly higher number of BODIPY-C11^OX^-positive macrophages than LPS-treated control macrophages (**Figure 6B**). Moreover, addition of the lipid peroxidation inhibitor Ferrostatin-1 (Fer-1) effectively prevented the manifestation of this *L. infantum*-induced BODIPY-C11^OX^-positive macrophage population (**Figures 6A, B**), confirming the specificity of the observed lipid peroxidation signal. Kinetic analyses subsequently revealed that *L. infantum* infection provoked slight lipid peroxidation starting from 48h post-infection, which increased significantly after 72h and 96h in comparison with LPS-treated control macrophages (**Figure 6C**). Interestingly, measuring cellular ROS levels with CellROX revealed an opposite picture in which LPS treatment caused progressive ROS accumulation, while additional *L. infantum* infection instead provoked a decline in cellular ROS levels (**Figure 6D**). These combined BODIPY-C11^OX^ and CellROX observations thus revealed a specific increase of oxidative stress at lipid membranes, suggestive of ferroptosis. However, monitoring of plasma membrane permeabilization kinetics revealed that Fer-1 did not diminish nor delay *L. infantum*-induced cell death in LPS-primed BMDMs (**Figure 6E**). Thus, while BODIPY-C11^OX^ analyses showed that *L. infantum* induced lipid peroxidation, blocking this ferroptosis hallmark with Fer-1 did not improve cell viability. Considering the possibility that *L. infantum* did not induce sufficient lipid peroxidation levels to cause ferroptosis, we next used macrophages deficient for the antioxidant enzyme Glutathione Peroxidase 4 (GPX4) to boost cellular lipid peroxidation and subsequent ferroptosis. For this purpose, we crossed mice bearing LoxP-flanked *Gpx4* alleles (23) with mice expressing an Fcgr1-driven macrophage-specific Cre recombinase (24). BMDMs from these Gpx4^MacKO^ mice lacked the GPX4 protein in naïve as well as LPS-primed conditions (**Figure 6F**). Moreover, the absence of GPX4 indeed lowered the threshold for lipid peroxidation, as *L. infantum* infection in LPS-primed Gpx4^MacKO^ macrophages resulted in significantly more BODIPY-C11^OX^-positive cells than in LPS-primed Gpx4^FL/FL^ macrophages (**Figures 6G, H**). However, elevated *L. infantum*-induced lipid peroxidation in Gpx4^MacKO^ macrophages was not accompanied by an increased or accelerated plasma membrane permeabilization (**Figure 6I**), showing that the absence of GPX4 did not sensitize these cells to ferroptosis. Taken together, neither pharmacological inhibition of lipid peroxidation with Fer-1 nor genetic boosting of lipid peroxidation through GPX4 deletion altered the sensitivity of LPS-primed macrophages to *L. infantum*-induced cell death. These results show that *L. infantum* induces Fer-1-sensitive lipid peroxidation that does not impact on cell death execution, suggesting that *L. infantum* is capable of uncoupling lipid peroxidation from cytotoxicity in LPS-primed macrophages.

**Figure 6:**
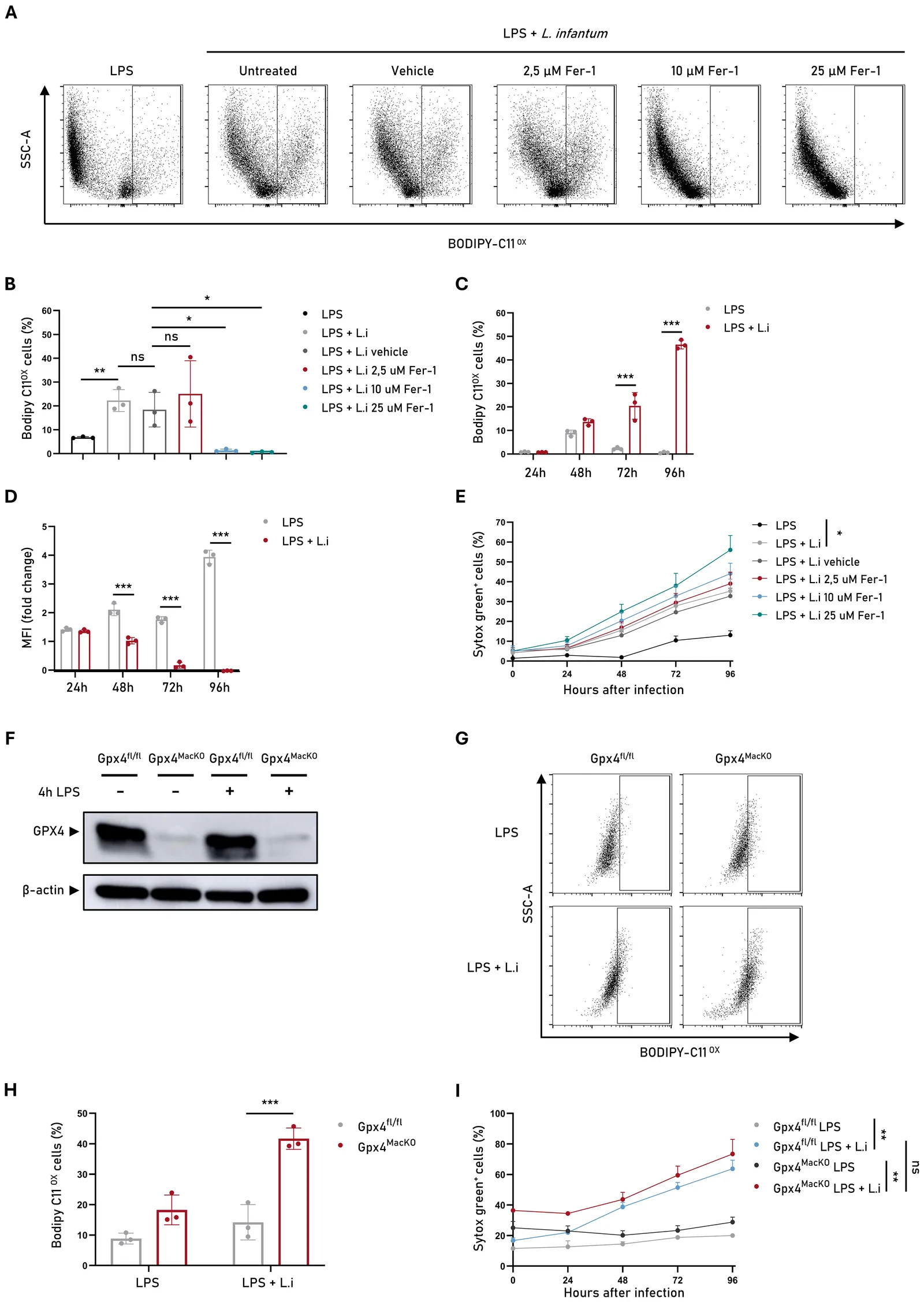
*Leishmania infantum* induces lipid peroxidation in LPS-primed macrophages independently of cell death. **(A–E)** Wild-type BMDMs (n=3) were treated with 100 ng/mL LPS for 4h and were then left non-infected or infected with MOI 10 of the LEM3323 WT^PpyRE9/DsRed^ *L. infantum* strain expressing DsRed for indicated time periods in the presence of SYTOX Green, and in the absence or presence of varying concentrations of Fer-1. **(A)** Representative flow cytometry plots and **(B)** quantification of C11-BODIPY^OX^ staining in the indicated setups at 96h post-infection. **(C)** Quantification of C11-BODIPY^OX^-positive cells at indicated timepoints and **(D)** fold induction of CellROX staining in infected BMDMs at indicated timepoints, relative to untreated cells. **(E)** Real-time imaging of SYTOX Green fluorescence showing cell permeability in the indicated setups. **(F)** Immunoblotting for GPX4 in Gpx4^FL/FL^ and Gpx4^MacKO^ BMDMs treated or not with 100 ng/mL LPS for 4h. **(G–I)** Gpx4^FL/FL^ and Gpx4^MacKO^ BMDMs (n=3) were treated with 100 ng/mL LPS for 4h and were then left non-infected or infected with MOI 10 of the LEM3323 WT^PpyRE9/DsRed^ *L. infantum* strain expressing DsRed for indicated time periods in the presence of SYTOX Green. **(G)** Representative flow cytometry plots and **(H)** quantification of plasma membrane C11-BODIPY staining in the indicated setups at 96h post-infection. **(I)** Quantification of SYTOX Green fluorescence showing cell permeability in the indicated setups. Data in **(B–E)** and **(H–I)** represent means ± SD of BMDMs taken from three individual mice per genotype. Statistics were analyzed with unpaired student t-test at 96 hours post infection **(B, E, H, I)**, or with two-way ANOVA **(C, D)**. ns, not significant; *p<0.05; **p<0.01; ***p<0.001.

Finally, we considered the possibility that ferroptosis collaborated with the other cell deathmodes in a redundant manner to facilitate macrophage death upon *L. infantum* infection. To examine this possibility, we treated *L. infantum*-infected LPS-primed Casp8^-/-^Ripk3^-/-^Casp1/11^-/-^ BMDMs with Fer-1. However, real-time Sytox Green imaging showed that Fer-1 did not alter plasma membrane permeability kinetics in these cells (**Supplementary Figure S2**), thereby indicating that combined inhibition of extrinsic apoptosis, RIPK3-dependent necroptosis, inflammasome-mediated pyroptosis, and ferroptosis did not prevent *L. infantum*-induced cell macrophage death, and suggesting that these cells succumb to another non-canonical cell death mode for which the execution mechanisms thus far remain elusive.

## Discussion

Viscerotropic *Leishmania* species such as *L. infantum* succeed in persisting inside the mononuclear phagocyte compartment of the liver, spleen and bone marrow through sophisticated strategies manipulating intracellular cell death signaling pathways as well as subverting host immune responses (4–6). In the context of *L. infantum* infection, the parasite was shown to employ several mechanisms for preventing macrophage apoptosis to survive in its primary intracellular niche. For instance, *L. infantum* infections in human macrophages differentiated from U-937 and THP-1 cell lines showed anti-apoptotic strategies including blockade of caspase-3 activation, initiation of the PI3K/Akt pro-survival pathway, and a transcriptional response leading to induction of anti-apoptotic factors such as A20 (33–35). Moreover, peripheral blood monocytes from VL patients display elevated Bcl2 expression, reflecting parasite-driven apoptosis resistance during human VL pathogenesis (36). Consistent with these parasitic strategies conferring host cell longevity even under heavy parasite loads, we observed that BMDMs started to display plasma membrane permeability indicative of cell death only from 72h post-infection despite efficient intracellular invasion of *L. infantum* already at 24h post-infection. Like in human U-937- and THP-1-derived macrophages, this suggests that *L. infantum* can slow down cell death of primary murine macrophages. Nevertheless, plasma membrane permeabilization at 96h post-infection indicated that *L. infantum*-infected BMDMs eventually were capable of initiating cytotoxic signaling pathways as a cellular anti-parasitic defense mechanism. In addition, we observed LDH release at 96h post-infection, showing that *L. infantum*-infected BMDMs underwent a lytic cell death mode. This lytic cell death implicates release of intracellular contents, including potentially viable parasites as well as host-derived damage-associated molecular patterns (DAMPs). As such, lytic cell death of infected macrophages could facilitate parasite dissemination to neighboring cells as was previously shown for *L. major* (37), and could as well influence nearby immune cells to amplify inflammatory host defense responses. Yet, secondary infection experiments as well as more thorough *in vivo* analyses are needed to determine whether the eventual outcome of macrophage lysis is advantageous for either the parasite or the host.

Interestingly, we show that the cytotoxic pathways triggered in *L. infantum*-infected BMDMs did not correspond to well-known cell death modes such as caspase-8 dependent extrinsic apoptosis, RIPK3-dependent necroptosis or inflammasome-instigated pyroptosis. Indeed, BMDMs incapable of launching these cell death programs due to combined genetic deficiencies in caspase-8, RIPK3, caspase-1 and caspase-11 displayed similar degrees of plasma membrane permeability upon *L. infantum* infection as wild-type BMDMs. The observed caspase-8-independent cell death appears to contrast with our immunoblotting result showing that wild-type BMDMs displayed proteolytic activation of caspase-8 from 24h and caspase-3 from 48h post-infection. However, the above-mentioned variety of anti-apoptotic strategies of the parasite render it plausible that *L. infantum* can suppress the execution of apoptosis downstream of caspase-8 and caspase-3 activation. Regarding RIPK3-mediated necroptosis, *L. infantum* was previously shown to trigger this lytic cell death mode in human and murine neutrophils upon inhibition of caspase-8 activity (38). When caspase-8 is inhibited, neutrophils initiate RIPK1-RIPK3 signaling, leading to necroptosis as a potent host defense mechanism associated with increased reactive oxygen species (ROS) production and significant parasite killing (38). In macrophages, pharmacological and genetic inhibition of RIPK1 kinase activity in human THP-1 macrophages and murine BMDMs, respectively, resulted in higher intracellular *L. infantum* loads (39). Although this study did not analyze cell death rates (39), it suggests that macrophages induce RIPK1 kinase activity-mediated necroptosis as an anti-parasitic strategy. Consequently, *L. infantum* may target necroptotic signaling to maintain intracellular replication in macrophages, which could explain why we did not observe RIPK3-dependent necroptosis in *L. infantum*-infected BMDMs. For instance, although supporting data specific for *L. infantum* in murine macrophages are lacking, *L. braziliensis* can counteract THP-1 macrophage necroptosis by diminishing both RIPK3 and MLKL expression levels (40).

Besides caspase-8 and RIPK3, our observation that *L. infantum* caused cell membrane permeability in Casp8^-/-^Ripk3^-/-^Casp1/11^-/-^ BMDMs showed that also caspase-1 and -11 were not involved in this process. This caspase-1 and -11 independency implies that *L. infantum* does not trigger canonical or non-canonical inflammasomes to induce cell death in BMDMs, which is in line with our observations that the parasite did not induce downstream GSDMD activation and that GSDMD deficiency did not impair *L. infantum* induced cell death. This inability of *L. infantum*-infected BMDMs to undergo inflammasome-induced GSDMD-mediated pyroptotic cell death aligns with a study showing that BMDMs infected with *L. amazonensis* showed no pyroptotic cell lysis (15). Mechanistically, this CL-causative *Leishmania* species induced non-canonical proteolytic processing of GSDMD, resulting in a 25kDa fragment that could not execute pyroptosis (15). However, we did not detect cleavage of GSDMD into a 25kDa fragment in BMDMs infected with the viscerotropic *L. infantum*. In addition, contrary to *L. amazonensis* that remained capable of inducing IL-1β secretion from BMDMs despite the absence of GSDMD-mediated canonical pore formation and pyroptotic lysis (15), *L. infantum*-infected BMDMs did not secrete detectable levels of IL-1β over a prolonged time period of 96h. Thus, while *L. amazonensis* is able to uncouple IL-1β secretion from pyroptosis, our results indicate that *L. infantum* employs a broader intracellular survival strategy avoiding both of these inflammasome signaling outputs in LPS-primed BMDMs.

Regardless of our *ex vivo* infection experiments in BMDMs arguing against a potent inflammasome-activating potential of *L. infantum*, elevated IL-1β levels in serum of *L. infantum*-infected mice suggest that inflammasomes do show a certain activity level during an *in vivo L. infantum* infection. In addition, intraperitoneal *L. infantum chagasi* infections in mice lacking ASC or caspase-1 showed that inflammasome signaling is protective (9), further supporting *in vivo* inflammasome activity and also suggesting a potential role for GSDMD in *L. infantum*-induced VL in mice. This discrepancy suggests that infecting *ex vivo* BMDMs with the parasite may not accurately reflect the host cell type or the parasite infection route that leads to physiological inflammasome activation in mice. For instance, regarding host cell type, mice with neutrophil-specific GSDMD deficiency exhibited increased parasite burdens upon a cutaneous *L. mexicana* infection (16). Thus, at least during *L. mexicana*-induced CL, neutrophils may represent a source for GSDMD-dependent IL-1β release. Alternatively, heterogeneity in the macrophage lineage itself may underlie varying regulation of inflammasome signaling. For instance, Kupffer cells are the initial host cells upon an intravenous *L. infantum* infection in mice. Infected Kupffer cells orchestrate granuloma formation, after which recruited monocyte-derived macrophages control parasite replication in later VL stages (28). The varying effector functions of these distinct macrophage populations may relate to differential signaling capabilities such as in inflammasome-induced pyroptosis, raising the possibility that particular organ- or VL stage-specific macrophage subsets could be responsible for *in vivo* IL-1β secretion during VL. Next, besides cell type, the parasite infection route is another variable, as *in vivo Leishmania* infection of macrophages happens subsequent to phagocytosis of infected apoptotic neutrophils (41). We currently cannot exclude that this physiological infection route through intercellular parasite transfer might be more relevant for triggering macrophage inflammasome signaling as compared to direct parasitic infections in *ex vivo* BMDMs. Taken together, caution is warranted when interpreting the physiological relevance of our observations in the reductionist BMDM infection model. For instance, it is not clear to which extent the altered transcriptional programs in LPS-primed BMDMs accurately reflect the activity status of macrophages infected in the host. Although macrophages *in vivo* may be exposed to a complex inflammatory environment, whether this environment functionally resembles the LPS-primed state of the BMDMs used in our study remains unknown. In addition, while we observed that *L. infantum* caused cell death in about 35% of LPS-primed BMDMs after 96h, this cell death frequency cannot be quantitatively extrapolated to *in vivo* infected macrophages. As such, the physiological significance of our BMDM findings for *in vivo* parasite dissemination or host immune responses currently is unclear. Nevertheless, we found that GSDMD-deficient mice overall did not display altered *L. infantum* infection kinetics or inflammation. Although an increased parasite burden was observed selectively at 4 wpi in the liver of Gsdmd^-/-^ mice, suggesting a transient local effect, our *in vivo* observations argue against a major role for GSDMD-dependent pyroptosis regulating an *L. infantum* infection. This contrasts with a study showing that GSDMD contributes to host resistance against *in vivo L. amazonensis*, *L. mexicana*, *L. major* and *L. braziliensis* infections (15), suggesting species-specific effects of GSDMD in modulating *Leishmania* infections in mice. Yet, mice infected with *L. infantum chagasi* showed Nlrp3-dependent IL-1β production that conferred parasite control (9). This apparent discrepancy with our observations in Gsdmd^-/-^ mice may relate to different experimental conditions, or may suggest that IL-1β release in infected mice occurs in a GSDMD-independent manner. In this respect, we did observe a minor upregulation of circulating IL-1β in serum of Gsdmd^-/-^ mice at 6 wpi. However, taken together, it is clear that significant amounts of *in vivo* research will still be needed to fully disentangle the complex *Leishmania* species-specific effects on GSDMD-mediated pyroptosis signaling in various organs and host cells, and at various stages during the infection.

Finally, we show that *L. infantum* induced robust lipid peroxidation in LPS-primed macrophages, but neither pharmacological inhibition of lipid peroxidation nor genetic sensitization via GPX4 deletion altered macrophage susceptibility to death. These observations may indicate that *L. infantum*-induced cell death in these macrophages does not qualify as *bona fide* ferroptosis, and as such could suggest that interfering with lipid peroxidation triggers a switch towards alternative non-ferroptotic cell death modes. Alternatively, our observations could indicate that *L. infantum* can uncouple lipid peroxidation from ferroptotic cell death in LPS-primed BMDMs. In support of this hypothesis, recent findings in murine peritoneal macrophages as well as in human monocyte-derived macrophages showed that *L. infantum* exploits cellular anti-ferroptotic defenses to escape macrophage-generated oxidative lipid peroxidation (42). In particular, activation of the transcription factor Nrf2 was shown to promote parasite survival in macrophages by limiting ROS and lipid peroxidation (42). Consistent with this redox-regulatory phenotype, we found that *L. infantum* infection downregulated cellular ROS levels at 72h and 96h post-infection when compared to LPS-treated control cells. This finding aligns with multiple studies demonstrating that *Leishmania* species evolved ROS-neutralizing strategies (43). However, Nrf2 activation was not directly assessed in our experimental model. Thus, while it is plausible that *L. infantum* in LPS-primed BMDMs also engages antioxidant pathways, potentially including Nrf2-driven responses, to maintain lipid peroxidation below the threshold required for ferroptosis, this remains to be experimentally established. Although we observed that GPX4 deletion did not suffice to lift lipid peroxidation levels above this crucial cytotoxic threshold, it is possible that disabling parallel anti-oxidant mechanisms such as ferroptosis suppressor protein 1 (FSP1) are additionally needed to effectively sensitize *L. infantum* infected BMDMs to this cell death mode. Indeed, FSP1 was shown to be capable of maintaining ferroptosis resistance even when GPX4 activity is compromised, in which combined disruption of these pathways was needed to sensitize to ferroptosis (44, 45). Other GPX4-independent ferroptosis-defense pathways have also been described, including the GCH1/BH4 pathway, which protects against lipid peroxidation through lipid remodeling and radical-trapping activity (46), and the mitochondrial DHODH/CoQH_2_ pathway, which can suppress mitochondrial lipid peroxidation in a context-dependent manner (47). However, we have not experimentally tested any of these additional GPX4-independent pathways in *L. infantum*-infected macrophages and therefore whether they contribute to the phenotype observed here remains unknown. Alternatively, instead of limiting the levels of lipid peroxidation, *L. infantum* may also preserve BMDM viability by affecting the subcellular membrane localization and propagation of the lipid peroxidation process. For instance, it was recently shown that initial compartmentalized lipid peroxidation within lysosomal membranes is required for subsequent spread to other cellular membranes in order to trigger ferroptotic cellular collapse (48). Although these possibilities remain to be tested, keeping lipid peroxidation levels below a ferroptosis execution threshold or spatially restricting this ferroptosis-driving process could constitute a complex host–parasite redox interplay through which *L. infantum* uncouples lipid peroxidation from cell death in infected macrophages.

In conclusion, our observations add considerable complexity to the multifaceted interaction between *L. infantum* and host cell death pathways. Our observations support prior studies showing that the parasite broadly suppresses cell death to maximize intracellular replication within long-term surviving macrophages (6). While *L. infantum* infection in macrophages was characterized by lipid peroxidation, neither blocking nor boosting this hallmark of ferroptosis impacted on parasite-induced cell death kinetics. This suggests that *L. infantum* can employ anti-oxidant strategies that uncouple lipid peroxidation from ferroptotic cell death. Nevertheless, we show that *L. infantum*-infected macrophages ultimately succumb through a cell death mode that is distinct from the major apoptotic, necroptotic and pyroptotic signaling pathways. However, the exact molecular execution mechanisms underlying this atypical macrophage death upon *L. infantum* infection currently remain unresolved. Future studies will be needed to identify the exact cell death pathways that are triggered by *L. infantum* within host macrophages during visceral leishmaniasis. As such, the immediate translational value of our work is complex and not yet clear from our present findings. Our study does not identify a single druggable pathway responsible for macrophage loss and we identify cell death and redox regulation as separable processes during *L. infantum* infection, which together suggests that therapeutic strategies manipulating either one of these processes may not be sufficient to interfere with parasite persistence inside macrophages.

## Data Availability

The original contributions presented in the study are included in the article/**Supplementary Material**. Further inquiries can be directed to the corresponding author.

## Supplementary material

Supplementary Figure 1 LPS-primed macrophages do not show inflammasome activity upon infection with *Leishmania infantum* at earlier timepoints.**(A–C)** Wild-type BMDMs (n=4) were treated with 100 ng/mL LPS for 4h and were then left non-infected or infected with MOI 10 of the LEM3323 WT^PpyRE9/DsRed^ *L. infantum* strain expressing DsRed for indicated time periods. **(A, B)** Cleavage of **(A)** GSDMD or **(B)** caspase-1 were assessed by immunoblotting. BMDMs treated with only LPS or with LPS/ATP served as negative control (NC) and positive control (PC), respectively. **(C)** IL-1β secretion was measured in cell supernatants at 24 and 48h post infection. Data in **(C)** represent means ± SD of BMDMs taken from four individual mice. Statistics were analyzed by unpaired student t-test, ns, not significant.

Supplementary Figure 2 *Leishmania infantum*-induced cell death in LPS-primed macrophages is independent of caspase-8, RIPK3, caspase-1 and -11, as well as lipid peroxidation. Casp1/11^-/-^Casp8^-/-^Ripk3^-/-^ BMDMs (n=3) were treated with 100 ng/mL LPS for 4h and were then left non-infected or infected with MOI 10 of the LEM3323 WT^PpyRE9/DsRed^ *L. infantum* strain expressing DsRed for indicated time periods in the presence of SYTOX Green, and in the absence or presence of 10 μM Fer-1. Quantification of SYTOX Green fluorescence showing cell permeability in the indicated setups. Data represent means ± SD of BMDMs taken from three individual mice. Statistics were analyzed by unpaired student t-test at 96h post infection, ns, not significant.
