# Supplementary material for: Leishmania infantum triggers lipid peroxidation and non-canonical cell death in macrophages
Source: Front Immunol. 2026 Oct 15;17:1891865. doi: 10.3389/fimmu.2026.1891865 (PMC13716562; doi:10.3389/fimmu.2026.1891865)
Supplement: Supplementary file 1 [file DataSheet1.pdf]

Figure S1.

A

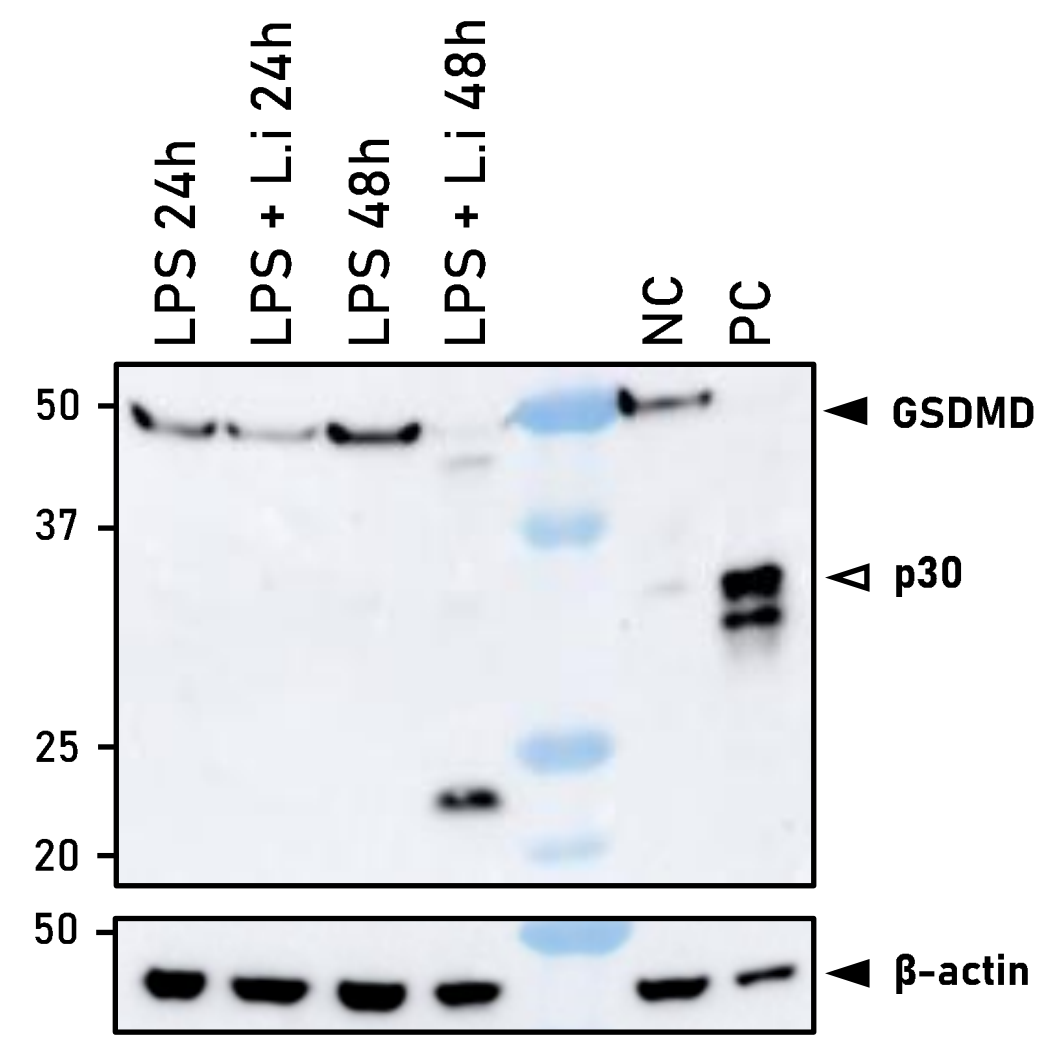

B

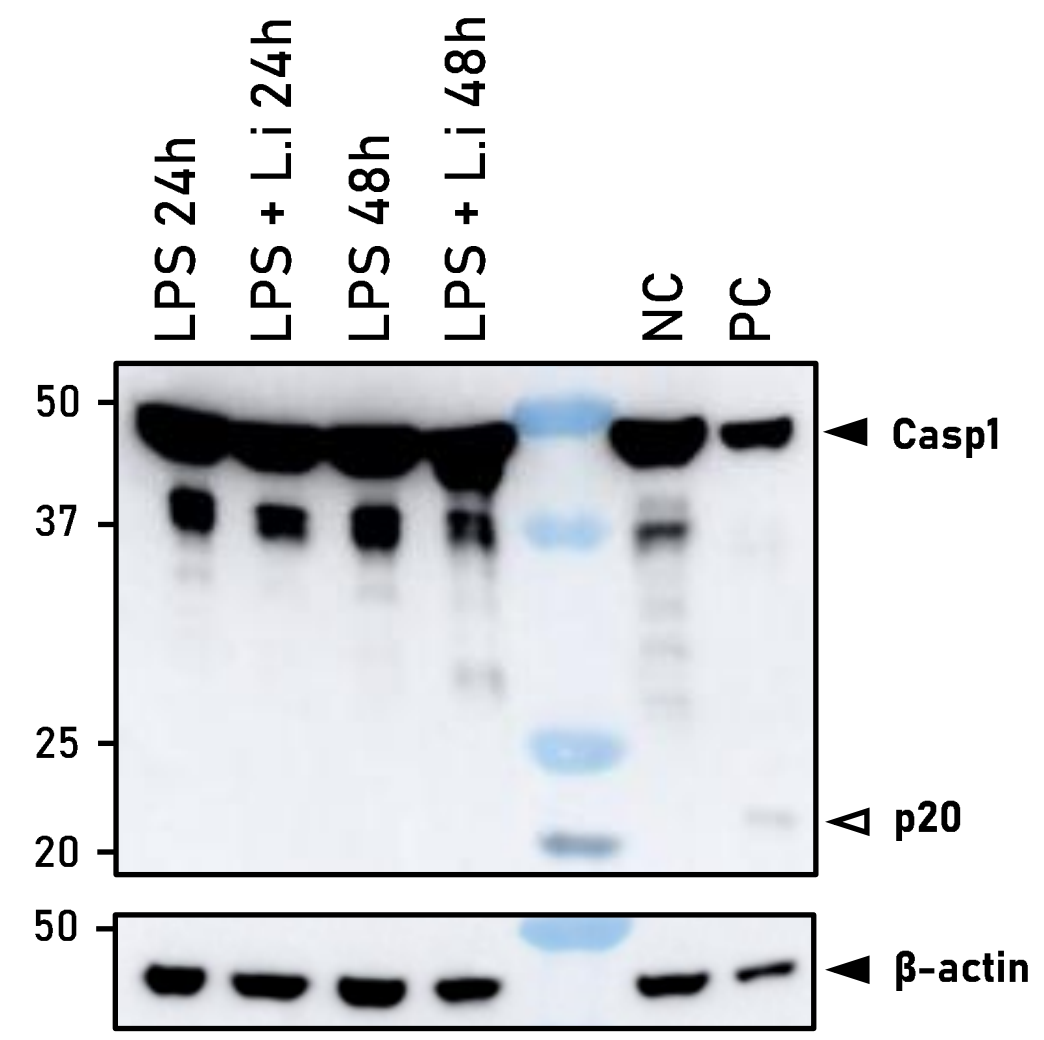

C

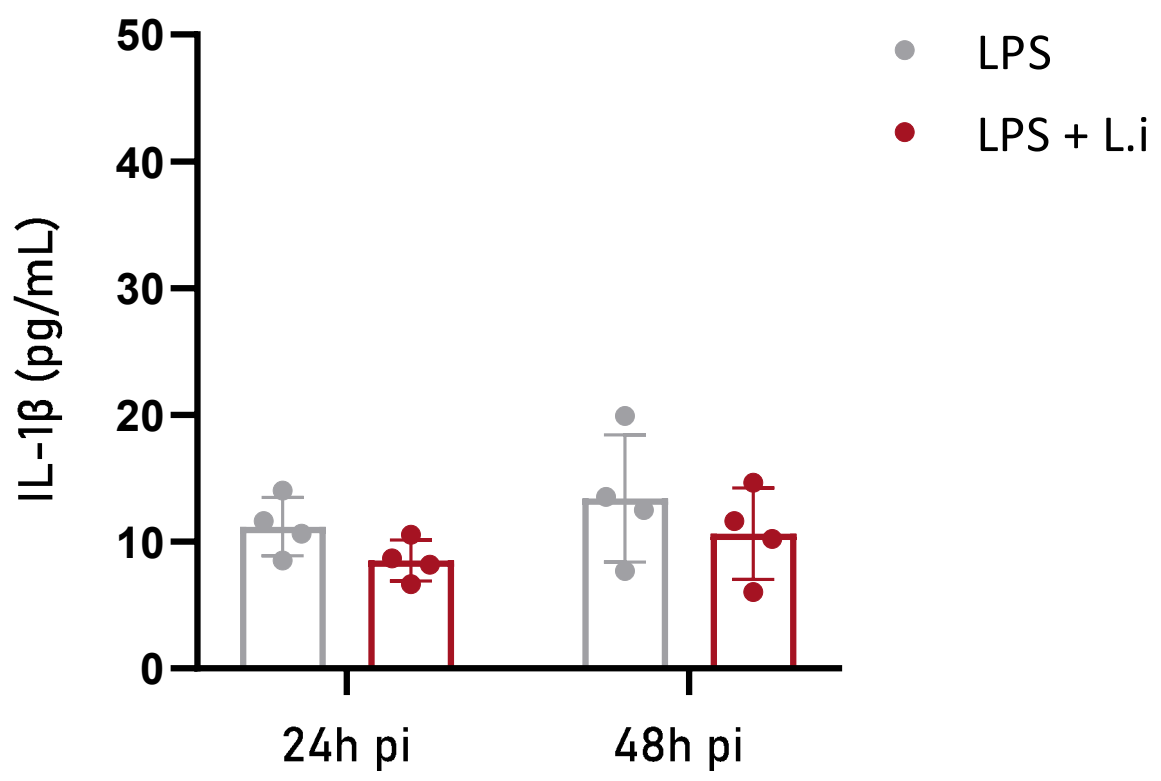

Figure S2.

A

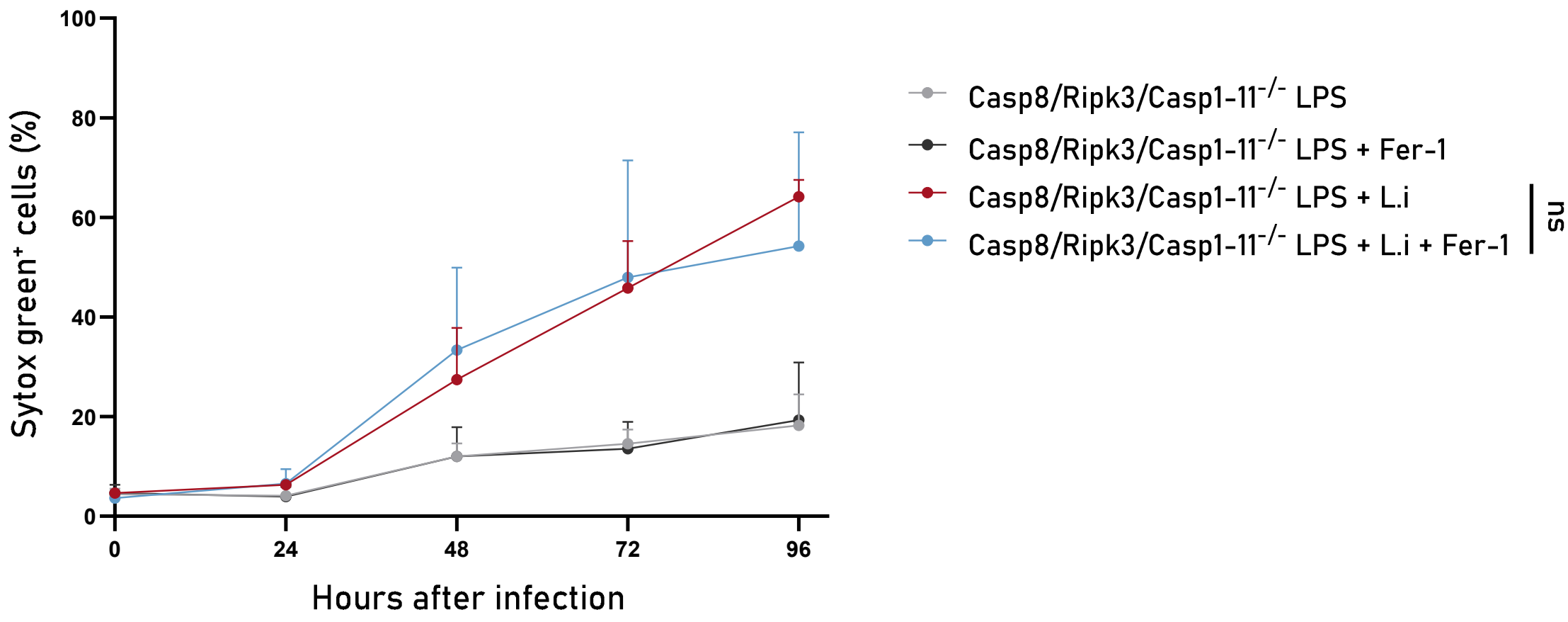

Sytox green+ cells (%)
100
80
60
40
20
0
0
24
48
72
96
Hours after infection
Casp8/Ripk3/Casp1-11-/- LPS
Casp8/Ripk3/Casp1-11-/- LPS + Fer-1
Casp8/Ripk3/Casp1-11-/- LPS + L.i
Casp8/Ripk3/Casp1-11-/- LPS + L.i + Fer-1
ns
